# Integration of Community Health and Prevention Topics in an Osteopathic Medical School Curriculum

**DOI:** 10.15694/mep.2020.000099.1

**Published:** 2020-05-13

**Authors:** Sara Goldgraben, Emily Johnston, Kristopher Bedi, Lisa Chun, Samuel Kadavakollu

**Affiliations:** 1 California Health Sciences University College of Osteopathic Medicine

**Keywords:** Community health, Prevention, Osteopathic medical school curriculum, Public health, COMLEX

## Abstract

This article was migrated. The article was marked as recommended.

Preventable chronic disease is a leading cause of death and disability in the United States and around the world. Training in wellness and disease prevention is provided to varying degrees in medical education leading to low levels of counseling on prevention-related topics in clinical care. Colleges of osteopathic medicine are in a unique position to lead the way in training future physicians in community health and prevention topics. Integrating community health and wellness throughout the curriculum allows osteopathic medical students to learn the scientific basis for prevention recommendations and learn to apply them to future patient care. We are incorporating these topics across the first two years of medical school in a new college of osteopathic medicine in the USA. This will address local disparities and train physicians prepared to care for patients at the individual and population health levels.

## Introduction

Personal health and wellness depend on modifiable and nonmodifiable factors. Healthy dietary choices, regular physical activity, adequate sleep, stress management, and avoidance of smoking can have positive long-term effects on health and the overall quality of life. Disease prevention and risk reduction strategies both at the clinical and public health levels can decrease the burden on the healthcare system, improve quality of life, reduce costs, and improve productivity ( Bauer *et al.*, 2014). In order to accomplish these goals, community health and prevention topics should be taught from the beginning of medical training for future physicians but are currently taught inconsistently throughout medical training in the United States. There is limited evidence that these gaps are made up during residency or specialty training, resulting in physicians inadequately prepared in preventive and lifestyle medicine and community health ( Krishnaswami *et al.*, 2018).

According to the National Board of Osteopathic Medical Examiners (NBOME) which administers the Comprehensive Osteopathic Medical Licensing Examination (COMLEX) that osteopathic medical students must pass in order to practice medicine, a minimum of 12% of questions are related to community health and wellness ( National Board of Osteopathic Medical Examiners, 2018). Integrating this material into the curriculum is not only good for the future of the healthcare system and quality of life of patients, but it is also an important preparation for these exams.

## Integration of Community Health and Prevention Topics

Preventive medicine and public health require attention not only to an individual’s choices of whether to engage in health promoting lifestyle activities, but also the family network they have, community resources available to them, support from public officials, and community partnerships. In California’s Central Valley where access to healthcare providers has historically been low and disparities high ( Conduent Healthy Communities Institute, 2020), it is important to take into account the social determinants of health, economic stability, education, social and community context, health and health care, neighborhood and built environment ( Office of Disease Prevention and Health Promotion, 2020). Some areas have limited access to healthy foods, an overabundance of unhealthy food options, limited paved roads, and sidewalks, making healthy lifestyle choices difficult. Further barriers physicians need to consider in caring for patients are things like lack of transportation that could limit the ability of patients to attend visits, lack of insurance or funds to fill prescriptions or to purchase and eat healthy foods; these all must be considered before patients are deemed noncompliant. The 2020 County Health Rankings from Robert Wood Johnson show that Fresno California scored 48 ^th^ place out of 58 counties for Health Outcomes and 51 ^st^ place out of 58 counties for Health Factors ( County Health Rankings, 2020). Colleges of osteopathic medicine should stay informed of the disease burden and disparities in their communities and accessing these types of data make it simple to perform a basic community health needs assessment and to plan programs to get medical students involved in reducing these disparities.

The purpose of this opinion piece is to demonstrate ways to incorporate these prevention topics into the medical school curriculum to better prepare future physicians that understand social determinants of health in an effort to reduce disparities in health outcomes. Introducing prevention topics in the medical school curriculum throughout the first two years will help to produce physicians who are aware of the challenges their patients face in the communities they live in. It will also give medical students tools with which to help patients prevent disease or prevent the advancement of disease early in life. As Benjamin Franklin famously said, “An ounce of prevention is worth a pound of cure.” In educating our medical students, who will then educate their patients, who will then share these lessons with their families, future generations can engage in healthy lifestyle habits and enjoy a better quality of life.

It is predicted that by 2025, there will be a shortage of over 50,000 primary care providers in the USA ( Nayyar *et al.*, 2018). Due in part to the lack of adequate primary care providers across the nation, community-level interventions are important prevention techniques that physicians can lead or contribute to. Colleges of osteopathic medicine often include care of underserved populations in their mission and tend to be placed in communities with low levels of access to primary care ( Ollove, 2014). In order to encourage future physicians to enter primary care fields and to train them in the prevention and public health, medical education must evolve. According to data from the American Association of Colleges of Osteopathic Medicine’s 2018-2019 Graduating Seniors Summary Report ( Research Department, 2019), 44% of participating students reported inadequate time devoted to instruction on cost-effective medical practice, 10% reported inadequate time devoted to health promotion and disease prevention, 36% reported inadequate time devoted to nutrition, and 14% reported inadequate time devoted to public health and community medicine. According to AAMC’s Medical Student Graduation Questionnaire 2019 All Schools Summary Report, 30.2% of respondents reported they plan to participate in public health activities in their careers ( Association of American Medical Colleges, 2019). Since 2015, greater than 25% of participating students have reported this. These reports show that medical students are interested in this knowledge and in pursuing careers that include public health and preventive medicine. The interest of students is apparent, and so is the need for this training as 40% or more of deaths from the leading five causes of death in the US are preventable ( Centers for Disease Control and Prevention, 2014).

The medical school curriculum is already packed with content, with limited room for adding new topics. Instead of creating a specific prevention curriculum, we have woven community health and prevention topics into the existing curriculum. For example, taking a history and physical is an essential part of the training of all physicians, and this can be applied to many prevention-related topics, including anything from intimate partner violence to seatbelt use ( **Table 1**). Offering new applications of traditional medical training topics allows for the updating of the medical school curriculum to meet the changing needs of society.

**Table 1.** Examples of curriculum areas where prevention topics can be integrated

Curriculum Area	Prevention Topic
**History Taking and Physical Exam**	Safety concerns-prevention, protection, and reporting of child, sexual, or elder abuse Intimate partner violence, sexual assault, gun safety or violation Environmental and public health risks Environmental screening Age-appropriate physicals for wellness, travel, sports, and employment Use of protective devices (i.e., helmets, seatbelts, infant/child seats)
**Immunology**	Age appropriate vaccinations Outbreaks/epidemics/pandemics/infection control/bioterrorism
**Musculoskeletal**	Fall prevention Ergonomics
**Ethics and End of Life Care**	Death related issues (i.e., right to die, medical futility, advanced directives) Coordination/transition of care
**Head and Neck**	Dental care Smoking cessation
**Cardiovascular**	Cardiovascular risk assessment (lipids, blood pressure, endocarditis prophylaxis) Diet assessment
**Pulmonary**	Smoking cessation Pollutants Coccidioidomycosis
**Renal**	Anticipatory guidance Blood pressure control Diabetes control
**Gastrointestinal**	Diet and exercise programs (obesity prevention)
**Endocrine**	Weight management, Diabetes Prevention Program referrals
**Reproduction**	Preconception and prenatal counseling Management of polycystic ovarian syndrome
**Neuroscience**	Environmental toxins screening (lead, secondhand smoke) Heavy metal poisoning
**Mechanism of Disease and Wellness**	Mental, physical, and spiritual health (stress management, diet and exercise programs, sleep hygiene, counseling) Motor vehicle operation safety
**Surgery**	Pre and postoperative procedure counseling Malnutrition and impact on recovery and outcomes
**Internal Medicine**	Pre and post-screening test counseling Genetic screening Medication safety (polypharmacy) Cancer screening
**Pediatrics**	Age-appropriate physicals for wellness Prevention of sudden unexpected death in infancy
**Psychiatry**	Substance abuse prevention and screening Management of medication-related weight gain/loss
**Obstetrics and Gynecology**	Prevention of sexually transmitted diseases Prenatal nutrition counseling

## Conclusion and Future Directions

Increasingly, medical schools are undertaking efforts to update their curricula and ensure it meets the changing healthcare needs of the population. It is imperative that these prevention and community health programs become lasting components in the curriculum after their pilot or short-course trials. These are novel curricular updates, and we will be collecting data along the way to make improvements and to ensure we are meeting the highest standards of osteopathic medical education. In conclusion, medical schools must remain adaptable to ensure they train future physicians to care for and to be aware of the changing needs of their patients and communities.

## Take Home Messages

• Adequate preparation of future physicians in preventive medicine topics is imperative to increase preventive health measures and reduce the burden of chronic disease in the US.
• There are many areas in the medical curriculum that can be enhanced by the addition of community health and prevention topics. Table 1 provides examples of how these topics can be incorporated.
• The licensing exam for students of osteopathic medicine (COMLEX) tests student knowledge of community health and wellness topics.
• Integrating this material into the medical school curriculum is not only good for the future of the healthcare system and quality of life of patients, but it is also important preparation for board exams.

## Notes On Contributors

**Sara Goldgraben**, MD, MPH, MBA, Assistant Professor of Specialty Medicine at California Health Sciences University College of Osteopathic Medicine.

**Emily Johnston**, PhD, MPH, RDN, CDE, Assistant Professor of Biomedical Education at California Health Sciences University College of Osteopathic Medicine.

**Kristopher Bedi**, DO, FACOG, Associate Professor and Chair of Specialty Medicine at California Health Sciences University College of Osteopathic Medicine.

**Lisa Chun**, DO, MS.MEdL, FNAOME, CPE, OHPE, Associate Professor and Associate Dean for Osteopathic Clinical Education at California Health Sciences University College of Osteopathic Medicine.

**Samuel Kadavakollu**, PhD, MSc, Associate Professor of Biomedical Education and Director of MCAT and Preparatory Programs at California Health Sciences University College of Osteopathic Medicine.
