# Supplementary material for: Integration of Community Health and Prevention Topics in an Osteopathic Medical School Curriculum
Source: MedEdPublish (2016). 2020 May 13;9:99. [Version 1] doi: 10.15694/mep.2020.000099.1 (PMC10697457; doi:10.15694/mep.2020.000099.1)
Supplement: Peer review history

# Reviewer report: Reviewer response for version 1

**DOI:** 10.21956/mep.20076.r30891

**Published:** 2020-10-13

**Authors:** Ken Masters

**Affiliations:** 1 Sultan Qaboos University

This review has been migrated. The reviewer awarded 4 stars out of 5 An interesting opinion piece on integrating Community Health and Prevention topics in an Osteopathic Medical School curriculum.The paper identifies the central problem that preventative medicine relating to personal health and wellness is taught inconsistently in the USA. These, and other psycho-social issues, need to be addressed in the curriculum.The authors also provide a useful starting framework for those who would consider incorporating some of the issues into their standard curriculum, in such a way so as not to overload the students. (This integration would also reduce the risk that the topic may be seen as an “extra” to the “real” medicine).As pointed out by the previous reviewer, the paper is more directly aimed at medical schools in the USA – to be fair to the authors, however, the paper does focus on the USA context; perhaps it might help if this were to be explicitly stated in the paper’s title.Nevertheless, a useful read for many who would like to have these issues addressed in the curriculum.

Reviewer Expertise:

NA

No decision status is available

# Reviewer report: Reviewer response for version 1

**DOI:** 10.21956/mep.20076.r30890

**Published:** 2020-06-11

**Authors:** Richard Hays

**Affiliations:** 1 James Cook University

This review has been migrated. The reviewer awarded 3 stars out of 5 I found myself in almost total agreement with the authors of this article, almost to the point of wondering if they are not stating the obvious, particularly to those of us engaged in community-based programs in areas of workforce and healthcare need. Their thinking is much more in keeping with the socially accountable medical education movement. I wonder if this message aimed at the USA medical education? For readers outside of the USA, more context might be helpful. For example, osteopathic schools are expanding and their graduates via the COMLEX process have recently been recognised as the equal of allopathic school graduates for all residency application purposes. Will the trend to family practice careers be disrupted by these changes? In other countries, embedding medical education in community-based programs has variable impact on career outcomes, with so many other influences pushing and pulling graduates into narrower specialties. More detail of future strategies and evaluation plans would have earned another star.

Reviewer Expertise:

NA

No decision status is available
